# Qualitative Research: Institutional Preparedness During Threats of Infectious Disease Outbreaks

**DOI:** 10.1155/2020/5861894

**Published:** 2020-01-23

**Authors:** Doret de Rooij, Evelien Belfroid, Renske Eilers, Dorothee Roßkamp, Corien Swaan, Aura Timen

**Affiliations:** ^1^National Institute for Public Health and the Environment (RIVM), Centre for Infectious Disease Control, Preparedness and Response Unit, Antonie van Leeuwenhoeklaan 9, Bilthoven 3721MA, Netherlands; ^2^Athena Institute, Free University, Amsterdam, Netherlands

## Abstract

**Background:**

As demonstrated during the global Ebola crisis of 2014–2016, healthcare institutions in high resource settings need support concerning preparedness during threats of infectious disease outbreaks. This study aimed to exploratively develop a standardized preparedness system to use during unfolding threats of severe infectious diseases.

**Methods:**

A qualitative three-step study among infectious disease prevention and control experts was performed. First, interviews (*n* = 5) were conducted to identify which factors trigger preparedness activities during an unfolding threat. Second, these triggers informed the design of a phased preparedness system which was tested in a focus group discussion (*n* = 5) were conducted to identify which factors trigger preparedness activities during an unfolding threat. Second, these triggers informed the design of a phased preparedness system which was tested in a focus group discussion (*n* = 5) were conducted to identify which factors trigger preparedness activities during an unfolding threat. Second, these triggers informed the design of a phased preparedness system which was tested in a focus group discussion (

**Results:**

Four preparedness phases were identified: preparedness phase green is a situation without the presence of the infectious disease threat that requires centralized care, anywhere in the world. Phase yellow is an outbreak in the world with some likelihood of imported cases. Phase orange is a realistic chance of an unexpected case within the country, or unrest developing among population or staff; phase red is cases admitted to hospitals in the country, potentially causing a shortage of resources. Specific preparedness activities included infection prevention, diagnostics, patient care, staff, and communication. Consensus was reached on the need for the development of a preparedness system and national coordination during threats.

**Conclusions:**

In this study, we developed a standardized system to support institutional preparedness during an increasing threat. Use of this system by both curative healthcare institutions and the (municipal) public health service, could help to effectively communicate and align preparedness activities during future threats of severe infectious diseases.

## 1. Background

The four pandemics (SARS, Influenza A/H1N1, MERS, Ebola) that have emerged since the beginning of this century [1] underpin the necessity of global awareness and optimal control strategies. These outbreaks showed the potential for the worldwide spread of such severe diseases [2] and led to social unrest and large economical consequences for the affected countries [3]. During the spread of the Ebola viral disease (EVD) outbreak in West Africa, the likelihood of imported cases in Europe increased, and European countries advised their healthcare institutions to prepare for patients with suspicion of EVD. Admission of a patient suspected for EVD, or another transmittable viral hemorrhagic fever (VHF), requires a large pool of trained healthcare workers and of specialized medical facilities [4, 5]. Therefore, in the Netherlands, the care for these patients was designated to a few highly specialized hospitals.

An evaluation by the Harvard-LSHTM Independent panel on the global response to ebola concluded that international response to EVD was inadequate [6]. A multidisciplinary national EVD outbreak evaluation in the Netherlands concluded that better guidance on preparedness during threats of outbreaks was needed for diseases, such as EVD, where patients only can be admitted to highly specialized hospitals [6]. In the Netherlands, as in other countries [7, 8], it had been unclear among curative and public health institutions which preparations during a developing threat of EVD were necessary, and for which preparations, the institutional or national level should have guidance [9].

While many studies describe preparedness for outbreaks, preparedness during a remote threat is not discussed separately in literature. The European Center for Disease Control defines preparedness for infectious diseases as “the knowledge and capacities […] to effectively anticipate, respond to, and recover from, the impacts of a likely, imminent or current crisis” [10]. Preparedness includes the development of institutional, national or international plans, communication and collaboration among different (types of) healthcare institutions, training and simulation, and surge capacity. The EVD outbreak, however, showed that successful preparedness during a threat requires flexibility and adaptations to be able to respond to differences in the probability of occurance of the disease. What these adaptations should be, has been unclear.

There is a need to clarify what preparedness entails in healthcare institutions during threats of severe infectious diseases whose patients can, due to the severity of the disease, only be admitted to designated, highly specialized hospitals (from now on described as diseases “which require centralized care”). Therefore, the aim of this study was to define preparedness during an unfolding threat of an infectious disease that requires centralized care. Second, we aimed to exploratively develop a standardized preparedness system describing preparedness activities for healthcare institutions in different preparedness phases.

## 2. Methods

**Figure 1:**
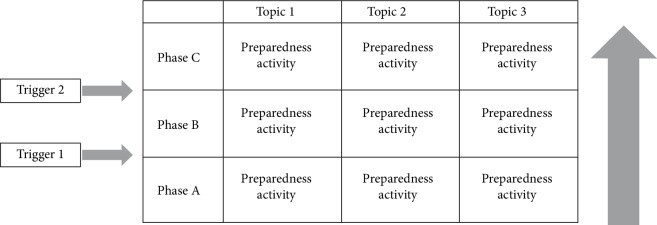
Concept preparedness system. The system consists of preparedness phases, defined by certain triggers; and by corresponding preparedness activities per phase, which are grouped per topic.

We conducted a qualitative three-step study with an iterative design of in-depth interviews (steps 1 and 3) and a focus group (step 2), in order to identify the key elements of a preparedness system. The system includes (a) the triggers for healthcare institutions to initiate extra preparedness activities during different levels of a threat, which define preparedness phases, and (b) preparedness activities for each preparedness phase. We aimed at finding generic triggers for preparedness, applicable to different types of healthcare institutions, such as hospitals, ambulance services, general practitioners and the municipal health services. The outline of the preparedness system is shown in Figure 1. In the first round of in-depth interviews, we explored the phases and triggers of the preparedness system, which we validated in the consensus meeting. In the second round of interviews, we aligned the preparedness activities per phase and grouped them per topic. We obtained ethical approval from the medical ethical committee of the UMC Utrecht (WAG/mb/17/028319). All participants provided informed consent and were informed that their responses would be used for research purposes.

### 2.1. Study Population and Recruitment

**Figure 2:**
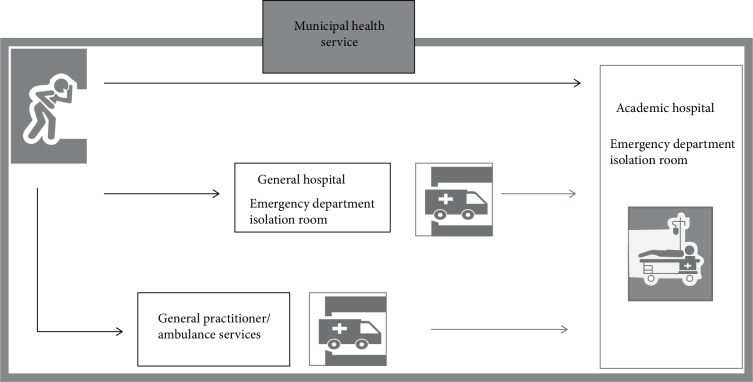
Healthcare institutions involved with a patient suspected for an infectious disease requiring centralized care. (Adapted from Swaan et al. [9].)

For all three steps, we invited professionals working at various levels and in various healthcare institutions and public health organizations. Included healthcare institutions were academic and peripheral hospitals, ambulance services, general practitioners, and municipal health services.  Figure 2 shows how healthcare institutions are involved in the case of a potential patient requiring centralized care [11]. The municipal health service (MHS) was additionally involved because of their coordinating role between all partners at the regional level. Professionals with the following backgrounds were invited:

- Academic hospitals: microbiologists and infectious disease specialists;
- Peripheral hospitals: infection preventionists;
- Ambulance services: medical managers at the regional and national level;
- General practitioners: respresentattives of the National Association for General Practitioners (LHV) and Dutch College of General Practitioners (NHG);
- Municipal health services: regional communicable disease control consultants and infectious disease control specialists.

We used purposeful sampling by approaching the key players in the Netherlands, from the above-mentioned healthcare and public health organizations, who were involved in preparedness and/or response during the EVD outbreak. When the invited key player was not able to participate, we asked him or her to nominate a colleague in the same type of healthcare institution with comparable expertise. All professionals had, in this way, expertise in EVD preparedness or response. For step 1 and 3 we aimed for one participant per healthcare institution. For step 2, we aimed for 1–3 participants per healthcare institution. Participants were invited by e-mail and a consecutive telephone call during September–November 2017.

### 2.2. Data Collection

Data collection comprised three steps: individual interviews, a focus group and another individual interview round. A hypothetical scenario of a VHF outbreak was used for data collection, which was developed by experts on outbreak control of the National Institute for Public Health and the Environment (RIVM). The scenario described a fictitious Marburg virus outbreak in Uganda that spread towards neighboring countries and continuously led to exported cases throughout the world. The outbreak scenario consisted of three stages, each hypothetically representing a preparedness phase of the preparedness system, as shown in Figure 1. Based on the identified triggers, preparedness phases for the preparedness system were developed. For each of these preparedness phases, again a corresponding scenario of the Marburg virus outbreak was developed to discuss in the focus group of step 2. The preparedness system was adjusted based on the results from step 2 and was sent by e-mail to the participant to discuss in step 3. The results of step 3 were used to finalize the preparedness system by grouping preparedness activities into overarching topics, and in institutional and collaborative activities.

#### 2.2.1. Step 1

Step 1 consisted of individual, in-depth, semi-structured interviews. To ensure that collected data was as reliable and consistent as possible, an interview guide was developed beforehand (additional [Supplementary-material supplementary-material-1]). The interview guide for step 1 was piloted with a microbiologist at a Dutch academic hospital. All interviews were conducted by one researcher (DdR), to safeguard inter-observer reliability. Interviews took between 35 and 50 minutes. During the interviews, the interviewer presented the outbreak scenario to the participant. Per preparedness phase, the participant was asked if and why preparedness activities would be necessary for their healthcare institution. In this way, the triggers for preparedness activities were explored. Alongside, questions covered terminology for preparedness during a threat, responsibility for preparedness during a threat, and collaborative preparedness activities carried out together with other healthcare institutions.

#### 2.2.2. Step 2

In step 2, a mixed focus group discussion with 1–3 representatives per type of healthcare institution was organized to validate the concept preparedness system. A focus group guide was developed beforehand (additional [Supplementary-material supplementary-material-1]). The focus group was guided by two researchers (DdR and CS), and supported by an expert in guiding focus groups (RE). The focus group took place at the RIVM and lasted 2 hours and 15 mintues. Per preparedness phase, the corresponding scenario was presented. Participants were asked if and why preparedness would be necessary within their institutions, what they would expect the other healthcare institutions to do, and where cooperation and support between healthcare institutions was needed. In the second phase of the focus group, preparedness activities identified in step 1 were presented to representatives of each type of healthcare institution separately. Representatives of one type of healthcare institution debated if and in which preparedness phase these preparedness activities were needed. Subsequently, terminology for preparedness during a threat was discussed among all participants, since the interpretation of terminology had shown to be different.

#### 2.2.3. Step 3

Step 3 consisted of individual, in-depth, semi-structured interviews. We included one participant per type of healthcare institution out of the participants in step 2. An interview guide was developed beforehand (additional file 1). Interviews were conducted by telephone and were all conducted by the same researcher (DdR). Interviews took between 15 and 20 minutes. The preparedness system was reviewed during the interview by discussing preparedness activities per phase. The participants discussed specific needs and adaptations per preparedness phase of the preparedness system. Further analyses included comparing preparedness activities between healthcare institutions, to see whether their expectations matched.

### 2.3. Data Analysis

Each interview and focus group was voice-recorded, with permission from the participants, and transcribed. Transcription started directly after the first interview and continued parallel to further data collection. Data were processed anonymously using a coding system. A summary of every interview and focus group was sent to the participants to verify their input. All interviews and focus group sessions were coded using content analysis. A coding guide (additional file 2) was developed beforehand, based on the structure of the interview guide. For each step, the guide was expanded and adapted. Coding was done by two researchers independently (DdR, and DR), using ATLAS.ti [12], and differences were discussed until consensus was reached. Data collected from each step of the study were analyzed and interpreted before the beginning of the subsequent step.

## 3. Results

### 3.1. Study Population

**Figure 3:**
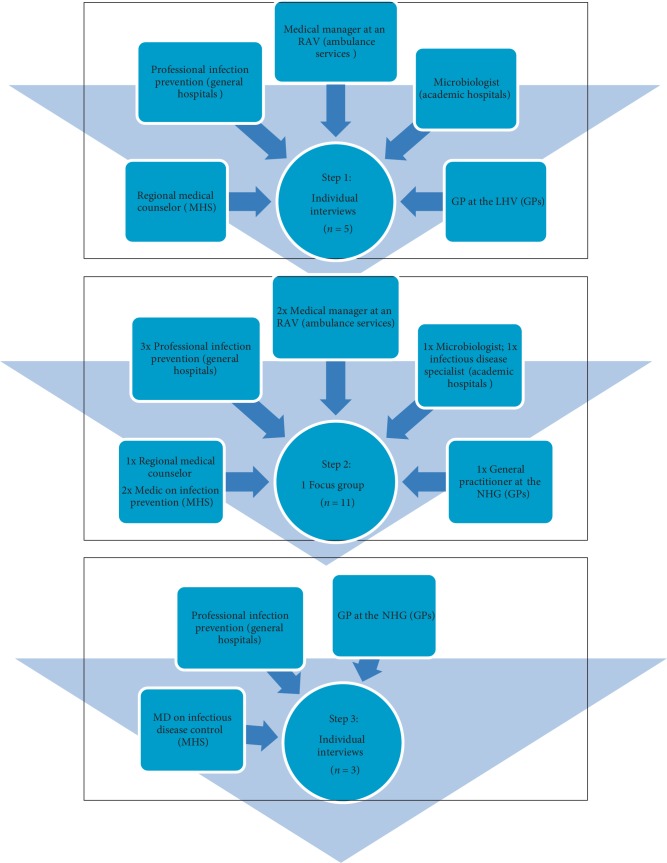
Included participants per step. N = number of participants, MD = medical doctor, RAV = region of ambulance services, MHS = municipal health service, GP = general practitioner, LHV = national association for general practitioners, NHG = dutch college of general practitioners.

In step 1, we invited 8 participants, of whom 3 could not be included. Five experts participated in the interview round: a microbiologist of an academic hospital, an infection preventionist in a general hospital, a medical manager of the national ambulance service with extensive experience as an ambulance nurse, a practicing GP and representative of the LHV, and a regional communicable disease control consultant of a municipal health service. In step 2, we invited 24 participants of whom 13 could not be included. Eleven experts participated in the focus group: 1 microbiologist and 1 infectious disease specialist of two different academic hospitals, 3 infection preventionists of different general hospitals, 2 medical managers of different regional ambulance services, 1 GP who also was representative of the NHG, and 3 regional communicable disease control consultants of different municipal health services. In step 3, we re-invited 7 participants from step 2, of whom only 3 accepted participation: one of the infection preventionists, the GP and representative of the NHG, and one of the regional communicable disease consultants. All representatives of academic hospitals and medical managers of the national ambulance services were either not responding or not able to participate due to time constraints. For all steps, reasons why professionals could not be included were the absence of reaction to the invitation (*n* = 7), unavailability during the data collection period (*n* = 11), completeness of inclusions (*n* = 2).  Figure 3 provides an overview of the different steps, the number of included participants and their backgrounds.

### 3.2. Terminology

During the first interview round, “scaling up” and “enhanced preparation” were used as synonyms by the interviewer for different preparedness phases. In the focus group, we observed differences in interpretation between curative and municipal health services. According to the curative partners, the Dutch term for upscaling that was used, applied to the response phase “with the presence of an actual potential patient”. In contrast, for the MHS, the term could also be used for preparedness during an increasing threat. The need for congruent language was highly stressed by the participants, and consensus was reached on the definition of *“enhanced preparedness”* to describe preparedness activities during a threat.

### 3.3. Triggers

During the first interview round several factors that trigger preparedness activities were identified for different healthcare institutions. The microbiologist at an academic hospital reported that they were at all times ready for such cases. However, they would enhance preparations if the likelihood of admitting a VHF patient increases, such as or with repatriated staff from the outbreak area. Municipal health services started with preparedness activities as soon as the outbreak somewhere in the world occurred and would be further enhanced when health institutions in their region were likely to become involved. For academic hospitals, general hospitals and ambulance services, (1) the likelihood that an unexpected potential patient was presented to their healthcare institution, and/or (2) unrest among the general population and staff, triggered preparedness activities. For general practitioners, only an outbreak in their would lead to preparedness activities.

In the focus group, trigger 1 and 2 were accepted as main triggers distinguishing between preparedness phases. Besides, a third trigger was the situation of several (potential) patients hospitalized within the country, conceivably leading to different referral pathways between healthcare institutions. Not all triggers would lead to the same intensity of extra preparations in all healthcare institutions, but all healthcare institutions would be involved in these phases. And most importantly, they all agreed upon the need for univocal communication.

**Figure 4:**
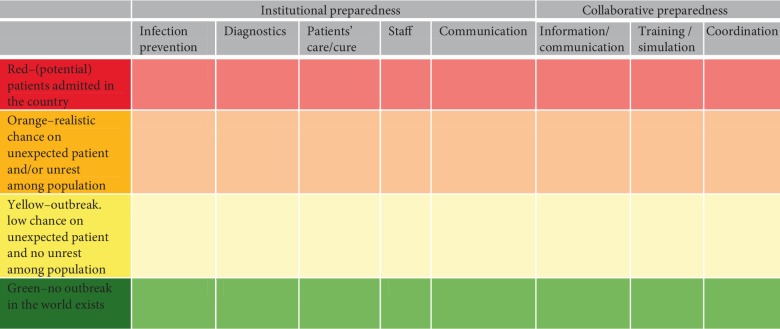
Preparedness system for threats of infectious diseases requiring centralized care. The system provides in rows the four ascending preparedness phase, and in columns the preparedness activities divided by content and grouped in institutional preparedness within or collaborative preparedness among healthcare institutions.

The final preparedness system based on these three triggers consists of four preparedness phases and is shown in Figure 4. Preparedness phase green is a situation without the presence of the infectious disease threat that requires centralized care, anywhere in the world. Preparedness phase yellow is the occurrence of the disease somewhere in the world but without triggers one and two. In preparedness phase orange, trigger one or two applies, and in preparedness phase red trigger number three applies.

### 3.4. Institutional Preparedness Activities

The preparedness activities as derived from step 1 and 2 and tested in step 2 and 3, were divided by topic as shown in the institutional preparedness column in Figure 4. All participants needed preparedness activities on infection prevention, such as the right type and stock of personal protective equipment, donning and doffing procedures, and waste management. Regarding diagnostics, academic hospitals described preparedness activities. These consisted mostly of extra checks whether differential diagnoses for these patients could run. Academic hospitals, peripheral hospitals and ambulance services named preparedness activities for patient care. Academic hospitals and peripheral hospitals discussed the need to prepare their personnel for extra working hours, or the need for extra supplies. General practitioners and municipal health services needed preparedness for controlling unrest among their staff and the population, e.g., by informing staff and the availability of a telephone line for questions. In additional file 3a, an overview of identified preparedness activities that resulted from step 2 and 3 are shown per type of healthcare institution. We identified the following trends:

- Academic hospitals start with all preparedness activities from preparedness phase yellow on. In preparedness phase orange, a sub-commission on preparedness for the admittance of patients needs to be installed, and in preparedness phase red, there will be a need to consider more ethical challenges related to the threat and challenges related to a shortage of staff.
- Peripheral hospitals inform triage staff and professionals at the gate during preparedness phase yellow. In preparedness phase orange, preparedness activities should start, except for diagnostics and patients' care/cure. In preparedness phase red, mainly a more intense communication among hospital departments and healthcare institution in the region is needed.
- For ambulance services, preparedness activities start in preparedness phase orange and no clear difference in preparedness activities were identified between preparedness phase orange and preparedness phase red.
- For general practitioners, phase orange is most important. The preparedness activities of general practitioners are limited to triage and primary infection prevention in all preparedness phases. Ethical considerations start in preparedness phase orange as well, but policy on this should ideally be made in the green phase.
- For MHS, diagnostics and training are required from preparedness phase yellow on, depending on the type of pathogen. In preparedness phase orange and red, they start to prepare their internal communication and personnel capacity.

### 3.5. Ethical Considerations

During step 1 and 2, ethical considerations were mentioned by representatives of all institutions except the municipal health services. The considerations of GPs, academic hospitals and ambulance services described how to deal with suspected cases in life-threatening situations. They need guidance on when concerns for their own safety would overrule their duty as a care provider, and based on which criteria. Another aspect named several times by representatives of ambulance services, general hospital and academic hospitals was the priority of care: to respectively transport, temporarily accommodate, or care for one EVD suspected patient, meant that many other patients could not receive care because an ambulance, an emergency department or large parts of intensive care had to close due to cleaning procedures, panic reduction or lack of staff or resources. Participants explicitly stated that these were dilemmas they faced during the latest EVD outbreak, and they would still face them should a case be admitted today.

### 3.6. Collaborative Preparedness

In addition to institutional preparedness activities, collaborative preparedness activities were discussed in all interview rounds. These are activities that overarch individual healthcare institutions or should be performed by multiple healthcare organizations together. We identified collaborative preparedness activities in information, training and simulation, and coordination, as shown in the columns headed “collaborative preparedness” in Figure 4. The expectations of the different types of healthcare institutions regarding information and coordination match well between healthcare institutions. This implies that information exchange between organisations is reported to be adequate. Furthermore, information on case definitions and information on the current preparedness phase is expected from the national centre for disease control. What did not match were the expectations of the different types of healthcare institutions regarding training and simulation exercises. The need to perform training or exercises together was mentioned by ambulance services and academic hospitals towards each other. But for peripheral hospitals, the need to practice together with ambulance services varied among participants. An overview of collaborative preparedness activities per preparedness phase is shown in additional file 3b. Participants of all types of healthcare institutions stressed that aligned preparedness activities are preferred over institutional autonomy. However, healthcare institutions with a specific function should be able to deviate from the preparedness system activities. Examples are healthcare institutions serving points of entry or those with national tasks such as the academic hospital with the reference laboratory.

## 4. Discussion

The aim of this study was to define preparedness during an unfolding threat of an infectious disease that requires centralized care. Second, we aimed to develop a standardized system describing preparedness activities per preparedness phase for healthcare institutions. We developed this standardized system by defining phases of preparedness during a threat and their corresponding preparedness activities, within both the perspective of individual healthcare institutions and of the collaborative network in which these institutions need to function. The four identified preparedness phases were based on (a) the likelihood of presentation of an infected patient to one of the healthcare institutions and (b) the unrest among the general population and staff. Phases ranged from no outbreak to the situation in which several potential or confirmed patients were hospitalized, conceivably leading to other referral pathways in the country. This system could be used for any future threat from an infectious disease requiring centralized care.

Using phases in preparedness to threats is not new. For terrorist attacks, for example, a level system using numbers 1–5 is common in several European countries, with 1 being considered a low threat, and 5 being a critical one [13]. In the Netherlands, a code system using colors is used in the weather forecasting, ranging from green (“business as usual”), through yellow and orange, to code red (“high impact on society”) [14]. And the WHO announced a pandemic phase system during the influenza outbreak (H1N1) in 2009, reaching from 0 to 6 [15]. However, by our knowledge, explicit preparedness phases following an unfolding threat caused by an infectious disease that offers concrete measures for frontline institutions have not been identified in literature. Certainly, we acknowledge the existence of the pandemic phases of the WHO [15]. These phases reflect the preparedness activities at a global, international and national level, rather than the institutional level within a country. The need for such specific phases for frontline institutions emerged during the evaluation of the Ebola threat [16], since all types of healthcare institutions experienced the need to perform extra activities to stepwise increase their level of operational response as the threat evolved. Healthcare institutions need thus to adapt their preparedness activities to the level of a threat.

The identified triggers for enhanced preparedness match with other studies and theory. Schol et al. identified higher fear among Dutch healthcare workers during the threat of Ebola and identifies a relation between fear and the need for information. This study provides support for our finding that unrest is a trigger for enhanced preparedness (in this case by providing additional information) [17]. The founding risk classification theory of Kinney and Wiruth (1976) [18]states that risk is the chance that something happens times the impact of that event. Within this formula, the presentation of an unexpected patient is the event that could happen, and the unrest among healthcare workers and the population represents impact. Together they define the risk, which is then translated in the urge to prepare. In this way, the phase system in this study builds upon the literature on risk classification.

Studies showed that extra preparedness was needed for countries during threats with increasing severity of outbreaks elsewhere in the world [7, 8, 19, 20]. However, these studies report on disease-specific preparedness activities and, therefore were, not necessarily applicable to other threats. This study used Marburg virus disease in the scenario and included experts with EVD outbreak experience. We worked in the aftermath of the EVD outbreak, but used a case of another disease. Hereby, we strongly aimed to work towards a generic preparedness system.

While specific preparedness activities differ between types of healthcare institutions and threat phases, in this study, a uniform enhanced preparedness system has been developed. During interviews, the focus group, healthcare institutions expressed the need to communicate explicitly and uniformly about preparedness activities. It became clear that there is no uniform terminology among experts from different healthcare institutions. For example, the term “scale-up” applies in curative care to the act of responding to an actual patient, while in public healthcare, the term could also be used during the preparedness. Absence of uniform terminology impedes communication between public and curative health care, while smooth communication between the two is a must, especially during threats or outbreaks. With clear definitions of phases, our system offers this uniformity both within institutions, as well as among institutions. It could therefore be used to effectively arrange communication about the required specific enhanced preparedness.

Although this specific study was conducted in the Netherlands, the results are also applicable in other countries with a comparable organization of healthcare. Centralized care in dedicated health centers for patients suspected for an infectious disease requiring centralized care, is described in Israel [7], the United States (New York State) [19], and in Canada [20]. Besides, they can be of value in other countries, because past experience with outbreaks has shown that presentation or even the likelihood of imported patients, indeed led to unrest among the general population and hospital staff [21, 22].

Our study has several limitations. There was a high attrition rate between the focus group and last interview round, leading to a lack of representation of academic hospitals and ambulance services. This has led to gaps in the completeness of the review of preparedness activities per stakeholer and per phase. However, to increase validity the outcomes of this study were presented and discussed in a 1,5-hour slot during the regular national meeting on EVD preparedness. During this meeting with national representatives of academic and peripheral hospitals, ambulance services and MHS who had been involved with preparedness and response during the EVD outbreak of 2013–15, the findings of this study were endorsed. This strongly supports further generalizability for both institutional as well as collaborative preparedness.

Another limitation is that data collection was only through interviewing, whereby direct observation of preparedness activities might yield additional findings. Also conducting a simulation exercise might lead to other insights. Moreover, we need to acknowledge the chance of recall bias. Although we used a new scenario, participants referred to activities they had performed two years before, during the EVD outbreak. This could have resulted in the identification of preparedness activities in phase yellow, orange and red that should not be performed in that phase. Participants might have reported from previous EVD experience where sometimes activities were performed in phase yellow, organge or red, whereas ideally these should be tackled in the green phase. An example are the ethical considerations, which indeed turn up during higher phases, but which should be covered in standard guidelines or procedures. An additional limitation is that most participants worked in the most urbanized parts of the Netherlands. Although regional organization might be different in the more rural regions, we have chosen to approach healthcare institutions with most experience with infectious diseases requiring centralized care. The expertise of the participants can be mentioned as a strength.

Since there was a strong need for a system that identifies different phases of a threat and the corresponding activities, this preparedness system could be used as a communication tool on a national or regional level. Future research should focus on identifying all activities for each phase. The completed preparedness system can be used by healthcare instiutions as a checklist of all preparedness activities they should perform during unfolding threats. In addition, it can be used as an agenda-setting for regional meetings to discuss the collaboration between healthcare institutions for unfolding threats. Finally, we recommend investigating the applicability of this system to other severe infectious diseases, not requiring centralized care. Examples could be, the recent outbreak of plague in Madagascar [23] or the ongoing threat of the Middle East Respiratory Syndrome-coronavirus [24]. Roles and responsibilities among types of healthcare institutions, in case of outbreaks of these diseases, vary and it is possible that other triggers and preparedness activities are required. Our phased preparedness system may also be applicable in these situations.

## 5. Conclusion

This study investigated preparedness during threats of infectious diseases requiring centralized care. This is the first study that explicitly defines preparedness activities during a threat for different frontline healthcare institutions. We reached consensus that a standardized preparedness system is required. A phased preparedness system has been developed, which can be used for improving institutional preparedness in curative healthcare institutions, and collaborative preparedness among curative healthcare institutions and the public health services.

## Data Availability

The datasets generated and/or analyzed during the current study are not publicly available due to the privacy protection of the participants, but are available from the corresponding author on reasonable request.

## Abbreviations

EVD: Ebola virus disease
GP: General practitioner
LHV: National association for general practitioners
MD: Medical doctor
MHS: Municipal health service
N: Number
NHG: Dutch college of general practitioners
PPE: Personal protective equipment
RAV: Region of ambulance services
RIVM: National institute for public health and the environment
VHF: Viral hemorrhagic fever
WHO: World Health Organization.

## Supplementary Materials

Supplementary Materials Additional File 1a: Guide for individual, semi-structured interviews, belonging to step 1 (written in Dutch). Additional File 1b: Guide for the mixed focus group, belonging to step 2 (written in Dutch). Additional File 1c: Guide for individual, semi-structured interviews, belonging to step 3 (written in Dutch). Additional File 2: Coding guide used for qualitative content analysis of data in step 1–3. (written in Dutch). Additional File 3a: Institutional preparedness activities per type of healthcare institution and per preparedness phase (written in Dutch). Additional File 3b: Collaborative Preparedness activities per preparedness phase.
