# Supplementary material for: Qualitative Research: Institutional Preparedness During Threats of Infectious Disease Outbreaks
Source: Biomed Res Int. 2020 Jan 23;2020:5861894. doi: 10.1155/2020/5861894 (PMC6998699; doi:10.1155/2020/5861894)
Supplement: Supplementary Materials — Additional File 1a: Guide for individual, semi-structured interviews, belonging to step 1 (written in Dutch). Additional File 1b: Guide for the mixed focus group, belonging to step 2 (written in Dutch). Additional File 1c: Guide for individual, semi-structured interviews, belonging to step 3 (written in Dutch). Additional File 2: Coding guide used for qualitative content analysis of data in step 1–3. (written in Dutch). Additional File 3a: Institutional preparedness activities per type of healthcare institution and per preparedness phase (written in Dutch). Additional File 3b: Collaborative Preparedness activities per preparedness phase. [file 5861894.f1.docx]

**Additional File 1a. Interview Guide Step 1 – Individual Interviews (Dutch)**

**Interview Guide voor individuele interviews**

Doel van het onderzoek:

Middels inventarisatie van het proces van verhoogde preparatie (kortweg ‘opschalen’) binnen de Nederlandse zorginstellingen (ziekenhuizen, huisartsen en ambulancezorg, GGD’en), komen tot een voorstel van standaardisatie van fasen en componenten van opschaling, welke aan het platform Groep A ziekten zal worden voorgelegd.

Aanwezigen:

- 1 interviewer, Doret de Rooij
- 1 deelnemer

Locatie:

De interviews zullen zo veel mogelijk in de professionele werkomgeving van de deelnemer plaatsvinden om de werkelijkheid zo dicht mogelijk te naderen.

Aanpak:

Potentiële deelnemers zijn via e-mail en telefonisch contact ingelicht over het onderzoek en gevraagd mee te doen. Voorafgaand aan het onderzoek zijn de informatiebrief en *informed consent* formulier naar de deelnemer opgestuurd. Bij deelname zal ruimte zijn voor vragen alvorens het *informed consent* formulier kan worden getekend. Er zal, bij nadrukkelijke toestemming van de deelnemer, gebruikt worden gemaakt van opnameapparatuur. Tijdens dit interview zal aan de hand van de presentatie van een casus de opschaling binnen de zorginstelling waar de deelnemer werkzaam is, worden besproken.

Tijdsplanning:

Informed consent en introductie 5 minuten

Definitie 5 minuten

Casus 1 - 3 20 minuten

Afschalen 5 minuten

Standaardisatie 5 minuten

Verhouding grootschalige uitbraken 5 minuten

Afsluiting 5 minuten

Benodigdheden:

voice recorder + extra batterijen, informatiebrief, informed consent formulier 2x, interview guide, pennen en kladpapier, LCI richtlijn/triagestandaard afh van de te interviewen partij, overzicht LCI/CIb, telefoonnummer geïnterviewde, onderzoeksvoorstel: hoofdvraag en nevenvragen.

Start

Bij aanvang van het onderzoek vraag ik of de deelnemer naar aanleiding van de informatiebrief en informed consent formulier nog vragen hebben, en als alle vragen beantwoord zijn en zij willen deelnemen vraag ik de deelnemer het informed consent te ondertekenen. Vervolgens zet ik, na toestemming van de deelnemer, de opnameapparatuur aan.

Introductie onderzoeker en onderzoek (5 minuten)

‘Mijn naam is Doret de Rooij. Ik doe de master Geneeskunde aan de Universiteit Utrecht, en loop nu een wetenschappelijke stage bij de LCI op het RIVM. U bent benaderd omdat u binnen uw zorginstelling een belangrijke rol speelt bij de voorbereiding en bestrijding van Groep-A-meldingsplichtige ziekten.

Ik wil graag weten wat opschalen bij een dreiging/opvang van een patiënt met een onder Groep-A-meldingingsplichtige virale hemorragische koorts concreet inhoudt. En daarom wil ik graag aan de hand van een casus hierover met u spreken in de komende drie kwartier.

Er zijn geen goede of foute antwoorden, ik ben benieuwd naar uw visie en expertise.

Hebt u nog vragen aan mij voordat we beginnen?

**Kunt u zich, allereerst, kort voorstellen en toelichten wat uw belangrijkste werkzaamheden zijn?**

**Kunt u ook aangeven wat uw werkzaamheden zijn op het gebied van de infectieziektepreventie?**

**Dank u wel, hoe zou u uitleggen wat het begrip ‘opschalen’ binnen de voorbereiding op een infectieziekte uitbraak inhoudt?**

Definitie/omschrijving

Belangrijkste aspecten

Betekenis in de dagelijkse praktijk

**Wat zijn uw ervaringen met het opschalen als gevolg van infectieziektenuitbraken of de dreiging daarvan?**

Algemeen infectieziekte-uitbraken
Virale hemorragische koorts/groep-A-ziekten

**U bent aan het werk en krijgt nu het volgende (inf@ct)bericht (door)gestuurd:**

**Inf@ct-bericht: Marburgvirusuitbraak in Kibale, Uganda**

In Kibale, in Zuidwest Uganda zijn in de afgelopen dagen drie patiënten gediagnosticeerd met Marburgvirus. De WHO coördineert de opvang en bestrijding. Op dit moment zitten 40 mensen, allen met Ugandese nationaliteit, in quarantaine.

**Wat doet u met een dergelijk bericht?**

- Zo ja, waarom? Hoe ziet dat eruit? Wat is het eerste wat u doet en wat vervolgens? Kunt u me meenemen in de processen en deze hardop uitspreken? (zie daarna de checklijst na de derde casus)
- Zo, nee, waarom niet? Wanneer wel?

**Dan ontvangt u op een dag het volgende bericht.**

**Inf@ct bericht: Marburgvirusuitbraak Uganda breidt zich uit**

Internationaal: In de afgelopen week heeft de Marburgvirusuitbraak zich uitgebreid tot nu 134 bevestigde gevallen. Naast verschillende steden in het westen van Uganda, zijn er nu ook tientallen gevallen bekend in Beni en Mbau, in het oosten van de DR Congo. De bron van de uitbraak is tot nu toe onbekend.

Nationaal: op dit moment bevinden zich tientallen Nederlanders (expats en toeristen) in het gebied, met wie op dit moment contact wordt gezocht. Er zijn nog geen Nederlandse gevallen bekend. De LCI onderstreept het belang dat ook Nederlandse zorginstellingen alert zijn op mogelijke gevallen onder terugkerende Nederlanders uit het gebied.

**Wat doet u met een dergelijk bericht? Zou u gaan opschalen?**

- Zo ja, waarom? Hoe ziet dat eruit? Wat is het eerste wat u doet en wat vervolgens? Kunt u me meenemen in de processen en deze hardop uitspreken? (zie daarna de checklijst na de derde casus)
- Zo, nee, waarom niet? Wanneer wel?

**Vervolgens ontvangt een volgend bericht**

**Inf@ct bericht: Marburgpatiënten in Duitsland en België**

Afgelopen week zijn er vijf bevestigde gevallen van Marburgvirus in Duitsland en België. Alle patiënten kwamen de afgelopen 5-10 terug uit Uganda of DR Congo. Twee patiënten hebben zich direct bij een spoedeisende hulp gemeld; drie patiënten zijn verwezen na een consult in de eerste lijn.

In Nederland zijn nog geen waarschijnlijke of bevestigde gevallen. De LCI vraagt artsen-microbiologen en deskundigen infectiepreventie nogmaals, alert te zijn, en om overige medewerkers binnen hun ziekenhuis, die betrokken kunnen zijn bij de opvang van een (verdachte) Marburgviruspatiënt, te informeren over ‘wat te doen’ bij de aankomst van een (verdachte) patiënt.

**Wat doet u met een dergelijk bericht? Zou u gaan opschalen?**

- Zo ja, waarom? Hoe ziet dat eruit? Wat is het eerste wat u doet en wat vervolgens? Kunt u me meenemen in de processen en deze hardop uitspreken? (zie daarna de checklijst na de derde casus)
- Zo, nee, waarom niet? Wanneer wel?

Checklijst:

- Wie bellen?
- Welke documenten raadplegen/richtlijnen volgen?
- Wat gaat er wezenlijk veranderen op de werkvloer?
- Wie is verantwoordelijk binnen de instelling en wat houdt dat in?
- Wie doet de werkelijke leiding? Wie initieert dit, wiens taak is dit?
- Wat is uw eigen rol?
- Welke mensen/organisaties zou u consulteren/samenwerking mee zoeken?
- Wat zou u op dit moment van het RIVM /LCI willen weten/krijgen?

Evt. Suggesties doen naar:

- - PBM (persoonlijke beschermingsmiddelen)
  - Personele bezetting
  - Communicatie
  - Educatie en Training
  - Vrijmaken opvang/isolatieruimten
  - Laboratorium diagnostiek
  - Etc.

**Inf@ct bericht: Uitbraak Marbrugvirus Oost Afrika onder controle**

**Wanneer zou u gaan afschalen?**

Zijn er, en dan welke voorwaarden?

Bij wie ligt dat initiatief?

Rol CIb?

**Wat is uw ervaring met afschalen?**

Successen

Problemen

Verbeteringen

**Hebt u behoefte aan verduidelijking/standaardisatie bij het op- en afschalen? Door wie zou dat volgens u kunnen/moeten worden geregeld?**

Binnen eigen organisatie
Buiten eigen organisatie
Wat is daarin uw eigen rol?

**Hoe verhoudt dit op- en afschalen zich tot andere uitbraken, bijvoorbeeld grootschalige griepepidemieën of een uitbraak binnen het ziekenhuis?**

**Wilt u nog wat kwijt over dit thema wat nog niet is besproken?**

**Dit was het, ik dank u hartelijk voor uw input.**

**U ontvangt een samenvatting van dit gesprek en kunt daarop nog feedback geven voordat ik het ga analyseren. Mochten er bij het verwerken van dit interview voor mij nog onduidelijkheden blijken, hebt u er dan bezwaar tegen als ik u daarvoor nog benader? Liever via mail/telefoon?**

**Nogmaals hartelijk dank uw deelname.**

Voicerecorder stop.

**Vraag: snowball sampling voor de focusgroepsessie**

**Additional File 1b Focus group Guide Step 2 (Dutch)**

**Focusgroep leidraad**

Doel onderzoek: inzicht krijgen in het proces van opschaling binnen Nederlandse zorginstellingen vanwege verhoogd risico op de uitbraak van een A-ziekte. Aspecten die worden bestudeerd zijn de veranderingen op de werkvloer, veranderingen op bestuursniveau, contactmomenten binnen de zorginstelling, contactmomenten buiten de zorginstelling, onzekerheden en verbeterpunten tijdens dit proces.

Aanwezigen:

- 1-3 moderators, Doret de Rooij, Renske Eilers, Corien Swaan
- 11 deelnemers

Locatie:

Zaal 0.009, U gebouw, RIVM, te Bilthoven

Tijdschema:

14.00 uur inloop met koffie en thee
**14.30 – 16.30 uur focusgroep:**
14.30 – 14.40 introductie en voorstelronde
14.40 – 15.20 casus 3
15.20 – 15.15 fase – indeling en terminologie
**15.25 – 15.40 PAUZE**
15.40 – 16.10 Invullen schema
16.10 – 16.20 afschaling
16.20 – 16.30 afsluiting en einde

**Aanpak:**

Voorafgaand aan het onderzoek zijn de informatiebrief en informed consent formulier naar de deelnemers opgestuurd. Er zal, bij nadrukkelijke toestemming van de deelnemers, gebruikt worden gemaakt van opname apparatuur. Tijdens deze focusgroepsessie zal aan de hand van de presentatie van een casus de opschaling worden doorgenomen.

**Benodigdheden:**

- Voice recorder + extra batterijen
- Interviewguide 2-3x geprint
- Dia’s uitgeprint 2-3x
- Lijst met te behandelen aspecten na de pauze
- Minstens 11 lege *informed consent* formulieren
- Minstens 11 pennen
- Minstens 11 x kladpapier
- Post its in minstens 6 kleuren, van elke kleur drie pakjes
- Beamer + flipovers
- Powerpointpresentatie op USB
- Digitaal format om mee te typen op het scherm op de USB
- Casus op USB
- Iedere casus minstens 11x uitgeprint
- Reiskostenformulieren 11x uitgeprint
- Naambordjes

**Tafelschikking**

Deelnemers van dezelfde keten naast elkaar zetten zodat zij ook onderling kunnen overleggen.

**Start**

Bij aanvang van de focusgroep vraag ik of de deelnemers naar aanleiding van de informatiebrief en informed consent formulier nog vragen hebben, en als alle vragen beantwoord zijn en zij willen deelnemen vraag ik de deelnemers te tekenen. Vervolgens zet ik, na toestemming, de opname apparatuur aan.

[start opnameapparatuur]

**Slide 1**

Welkom allemaal, en wat leuk dat jullie in grote getalen aanwezig zijn. Voor jullie staan Renske Eilers, onderzoeker bij de LCI, Doret de Rooij, master geneeskundestudent uit Utrecht. En ik ben Corien Swaan hoofd van de afdeling Preventie bestrijding en crisismanagement en voorzitter van het platform preparatie Groep A ziekten.

Wat is het doel van vandaag? Wij proberen een opschalingssystematiek te ontwikkelen voor in de voorbereidende fase bij een dreiging van een ernstige virale hemorragische koorts of andere groep A ziekte die centralisatie van de opvang van patiënten vereisen. Uit de ebolaevaluatie na de ebolacrisis in 2014-15 kwam naar voren dat het voor ketenpartners onduidelijk was wat opschaling in de voorbereidende fase inhield, en daar werd om duidelijkheid gevraagd.

De focus ligt op de institutionele preparedness van de curatieve zorg. Dat zijn de aanwezigen van de academische en perifere ziekenhuizen en ambulancezorg. We zijn erg blij dat ook vanuit de publieke gezondheidszorg en huisartsgeneeskunde mensen aanwezig zijn, omdat hun rol in de opschaling en dus de aansluiting naar deze ketenpartners van groot belang is.

Er heeft in dit onderzoek al een eerste ronde van individuele interviews plaatsgevonden. De resultaten daarvan gebruiken wij bij u vandaag om verder onderzoek te doen.

**Slide 2**

Vandaag gaan we verschillende dingen doen. Het eerste deel van vanmiddag zal zijn dat een aantal scenario’s van een casus doorlopen en u vragen om vanuit uw expertise en instelling wat u naar aanleiding van dat bericht gaat doen. Daarna bespreken een voorstel van een opschalingssystematiek en willen we dat met u invullen. Ten slotte zullen we het ook nog over afschalen hebben.

Zijn hier tot zover vragen over?

**Slide 3**

Dan lijkt het mij goed om eerst een korte voorstelronde te doen.

[voorstelronde]

**Toetsen inhoud van de verschillende fasen**

Dan wil ik nu beginnen met het eerste deel van de casus.

**Slide 4**

**Bericht 1 –**

In de afgelopen weken breidt een uitbraak van het Marburgvirus zich verder uit in het zuidwesten van Uganda, en in het aangrenzende oosten van Congo. De WHO coördineert de opvang en bestrijding. Er zijn tot nu toe 40 bevestigde gevallen. 350 mensen verblijven in quarantaine, onder hen vijf internationale hulpverleners.

Er bevinden zich een tiental Nederlandse expats en hulpverleners in het gebied met wie op dit moment contact wordt gezocht. Enkele Nederlandse artsen van een medische NGO zijn afgelopen week afgereisd om te ondersteunen.

De LCI bereidt voor een eventuele repatriëring van een (verdachte) patiënt via de medische NGO of een expat, via BuZa/VWS.

Dit bericht komt van een betrouwbare bron, en komt u tijdens uw werk onder ogen. Lees het bericht even rustig door.

De vraag aan u is wat dit bericht voor u als professional en binnen de instelling waar u werkt betekent. Wat gaat u nu doen?

Wie wil er reageren? **Iemand typt mee op het scherm**

Suggesties:

- Wie inlichten? Binnen/buiten eigen instelling
- Wie/wat raadplegen? Binnen/buiten eigen instelling
- Verandert er iets op de werkvloer? Wat dan?
- Wat heb je nodig? Van wie binnen/buiten eigen instelling?
- Onderwijzen/ oefen/trainen? Met wie binnen/buiten eigen instelling
- Inkoop?

Ondertussen mensen op elkaar laten reageren, laten aanvullen.

Wat als er nog 10 toeristen nog een week in het gebied verblijven?

**Slide 5**

Een dikke week later volgt het volgende bericht. Lees het weer even rustig door.

**Bericht 2 –**

Afgelopen weken heeft de uitbraak zich verder verspreid naar Congo, Rwanda en Tanzania. De WHO geeft een wereldwijde waarschuwing af (PHEIC). Wereldwijd zijn meerdere verdachte patiënten opgevangen. In Canada en Israël zijn twee gevallen bevestigd.

In Nederland zijn 2 potentiele gevallen opgenomen, die bleken bij nader onderzoek malaria te hebben. Niet noodzakelijke reizen worden ontraden, maar er is geen negatief reisadvies

De LCI vraagt Nederlandse ziekenhuizen, ambulancezorg, GGD en HA alert te zijn en zich voor te bereiden op de mogelijke komst van een (verdachte) patiënt in Nederland.

[lezen]

Wat verandert dit. Wat gaat u nu doen? **Iemand typt mee op het scherm**

Wie wil er reageren?

Suggesties:

- Wie inlichten? Binnen/buiten eigen instelling
- Wie/wat raadplegen? Binnen/buiten eigen instelling
- Verandert er iets op de werkvloer? Wat dan?
- Wat heb je nodig? Van wie binnen/buiten eigen instelling?
- Onderwijzen/ oefen/trainen? Met wie binnen/buiten eigen instelling
- Inkoop?

**Slide 6**

Ten slotte volgt nog dit bericht. Lees het weer even rustig door.

**Bericht 3 –**

In Nederland liggen 4 mensen met bevestigde diagnose Marburgvirus opgenomen, en er zijn 2 potentiële patiënten in onderzoek, in vier Nederlandse UMC’s

Wat gaat er nu gebeuren? **Iemand typt mee op het scherm**

Suggesties:

- Wie inlichten? Binnen/buiten eigen instelling
- Wie/wat raadplegen? Binnen/buiten eigen instelling
- Verandert er iets op de werkvloer? Wat dan?
- Wat heb je nodig? Van wie binnen/buiten eigen instelling?
- Onderwijzen/ oefen/trainen? Met wie binnen/buiten eigen instelling
- Inkoop?

**Toetsen Indeling en criteria van fasen.**

**Slide 7**

Wat jullie hier zien is een voorstel van opschalingssystematiek, beschreven in drie fasen. Wat we zojuist hebben gedaan zijn scenario’s besproken van dit voorstel van een opschalingssystematiek. De voorwaarden tussen de fase komt voort uit de eerste interviewronde. Wat bleek namelijk, is dat de kans dat zich onverwacht mensen presenteren bij alle partijen bleek te bepalen welke maatregelen werden genomen. Op basis hiervan hebben we dus fase 1 en fase 2 benoemd.

Aan de hand van uw input net, uw reacties op de casus kunnen we dit schema verder invullen. Er staan nu in de bovenste rij wat voorbeeld op basis waarvan dit schema dan wordt ingevuld. En dit is het voorstel van het schema wat wij willen ontwikkelen en wat hopelijk ook bruikbaar is om inzicht te krijgen in wat iedere ketenpartner in welke situatie doet en verwacht. En wat hopelijk de communicatie en verwachtingen ook faciliteert.

**Wat vindt u van deze indeling, en van deze systematiek?**

**[input]**

**Blijf bij slide 7**

**Toetsen terminologie**

**Hoe zouden jullie dit proces / de verschillende fasen noemen? /**

Suggesties: Hebben we het hier volgens u over opschaling? Of over verhoogde preparatie etc.

[Doel: Middels input tot andere naam komen.]

**PAUZE**

**Toetsen van de aspecten van opschaling – interactief**

Er zijn al heel wat aspecten aan bod gekomen tijdens het eerste uur. Wat we nu willen doen is u nog wat voorstellen doen van dingen die mogelijk belangrijk zijn tijdens de opschaling. Dit zijn aspecten die wij hebben gehaald uit de literatuur en uit de eerste ronde van dit interview. En wij zouden graag uw mening horen over of dit in dit schema thuishoort, waar dan, en wat specifiek. Dat is nogal een opdracht en dat willen we dus op de volgende wijze doen.

Iedere ketenpartner krijgt een stapeltje post its in een eigen kleur. En er hangen hier vier bladen die de vier fasen representeren die wij zojuist overeen zijn gekomen. Van boven naar beneden staan wat onderwerpen (aspecten) die naar onze mening nog onvoldoende aan bod zijn gekomen voor de pauze. Aan u de vraag of u een post it van uw kleur wil plakken achter het onderwerp (aspect) dat tijdens een bepaalde fase aandacht verdient. De kleur zegt in principe al voldoende. Maar u mag aanvullend die post it schrijven wat u precies zou willen zien. Het mooiste is als u het binnen uw keten eens bent en gezamenlijk een post it plakt, maar als dat niet zo is, wees ook vrij om individueel bijdragen te doen, maar schrijf dan uw naam erop.

Bovendien mocht u nog wat willen toevoegen maar past dat niet binnen de categorieën dan mag u dat ook op een post it schrijven en onder de fase plakken.

[Voorbeeld benoemen.]

Is dat duidelijk voor iedereen? Zijn er nog vragen?

**[tijd om te plakken en te schrijven]**

**Afschaling**

Goed, als iedereen weer wil gaan zitten. Ten slotte, bij opschalen [of nieuwe term] hoort ook weer afschaling. Uit de eerste ronde blijkt dat het moment van afschaling onduidelijk was. Het belang van een landelijke congruente afschaling binnen ketenpartners als wel tussen ketenpartners wordt van belang geacht. De LCI wordt aangewezen als belangrijke maat/raadgever daarin en wordt verzocht concreet te zijn. Aan de andere kant geven ketenpartners aan zelf de regie te hebben en willen houden. De LCI wil ook niet de gehele verantwoordelijkheid dragen want wil ook niet dat iedereen ineens niets meer doet.

**DISCUSSIE**

**Afsluiting**

- **Samenvatting wordt opgestuurd**
- **Presentatatie platform A ziekte**
- **DANK!**

**Additional File 1c Interview Guide Step 3 (Dutch)**

**Interviewguide bij interviewronde 2**

Gevraagde tijd: 20 minuten

Aanpak: Per mail respondent focusgroep benaderd middels ‘purposeful sampling’ waarbij uitnodigen voor (telefonisch) vervolg gesprek.

Ethische afwegingen: Respondenten worden geïnformeerd dat de data hetzelfde zullen worden behandeld als tijdens de focusgroep en dat hetzelfde informed consent geldt. Dat we ook dit gesprek graag zouden opnemen. Bij mondelinge toezegging wordt dat gedaan.

**Bijlagen in de mail:**

1. Faseoverzicht:

Fase 1: Uitbraak ver weg van ziekte die centralisatie van patiënten vereist, zeer lage kans op onverwachte (potentiële) patiënten in Nederland, mogelijke repatriëring via de daartoe bestemde kanalen, die leidt tot weinig onrust onder de bevolking.

Fase 2: Uitbraak ver weg van ziekte die centralisatie van patiënten vereist, aannemelijke kans dat onverwacht (potentiële) patiënten in Nederland zich presenteren naast mogelijke repatriëringen, mogelijk onrust onder de bevolking en/of personeel op de werkvloer.

Fase 3: Uitbraak van ziekte die centralisatie van patiënten vereist en heeft geleid tot importgevallen in Nederland waardoor er moet worden ingespeeld op voldoende regionale en nationale capaciteit voor de opvang en behandeling.

1. Fase-indeling per betrokken ketenpartner + door henzelf genoemde aspecten per fase

**Bij het interview:**

Danken voor deelname.

**Dubbelcheck dat ik mag opnemen. [start opname]**

Ruimte geven om eerste indruk aan de hand van de samenvatting te geven

Begrijpelijkheid van de bijlagen toetsen.

Begin ik met de eerste vraag. Vanaf welk punt voor u fase 3 begint, en dus ofwel een capaciteitsprobleem zich voordoet of buiten de geoefende lijnen moet worden opgetreden?

[…]

Dan de tweede vraag: Kunt u per fase aangeven met welke ketenpartners u contact hebt, wie van u wat nodig hebt, of van wie u wat nodig hebt? Laten we beginnen bij fase 1.

[…]

En in fase 2?

[…]

En in fase 3?

[…]

Tenslotte heb ik nog het overzicht bijgevoegd het overzicht van acties en benodigdheden zoals u dat hebt ingevuld middels de post its, tevens in de bijlage te vinden. Bent u het nog steeds eens met hoe dit is ingevuld?

[…]

Ik had zelf nog een specifieke vraag over…

Wilt u zelf nog wat toevoegen? […]

Nogmaals dank voor deelname. De resultaten zullen worden gepresenteerd tijdens de platformbijeenkomst en ook aan u worden toegezonden.

[stop opname]

**Additional File 2. Coding Guide (Dutch)**

**Definitie opschaling**

- 1. definitie opschaling – algemeen

1.2 educatie (training)

1.4 controleren

1.5 informeren (informatie)

1.6 capaciteit

1.7 aanwezigheid patiënt

1.8 impact

1.9 capabiliteit

1.10 coordinatie

**2.0 binnen fase 0**

2.0.1 Infection control

*2.0.1.2 persoonlijke beschermingsmiddelen*

*2.0.1.2 veilige opvang*

*2.0.1.3 surveillance*

*2.0.1.4 aanwijzen opvang/behandelcentra*

2.0.2 Diagnosis

2.0.3 Patiënten zorg

*2.0.3.1 behandelprocedures*

2.0.4 Staf/mankracht

2.0.5 Institutionele capaciteit

*2.0.5.1 beschikbaarheid isolatiekamer*

2.0.6 derden

*2.0.6.1 burgers*

2.0.7 trainen

*2.0.7.1 opvang*

*2.0.7.2 behandelen*

*2.0.7.3 overdracht*

*2.0.7.4 monsterafname*

*2.0.7.5 aan-uitkleden*

*2.0.7.6 overig/algemeen*

2.0.8 communicatie/informatie

*2.0.8.1 interne selecte communicatie*

*2.0.8.2 externe selecte communicatie*

*2.0.8.3 interne algemene communicatie*

*2.0.8.4 externe algemene communicatie*

2.0.9 ethiek

*2.0.9.1 levensreddende handelingen*

2.0.10 Overig/algemeen

**2.1 binnen fase 1**

2.1.1 Infection control

*2.1.1.1 triage*

*2.1.1.2 casusdefinitie*

*2.1.1.3 Persoonlijke beschermingsmiddelen (PBM)*

*2.1.1.4 afvalverwerking*

2.1.2 Diagnosis

2.1.3 Patienten zorg

*2.1.3.1 beleid*

2.1.4 Staf/mankracht

2.1.5 Institutionele capaciteit

*2.1.5.1 materialen*

2.1.6 derden

2.1.7 trainen

2.1.8 communicatie/informatie

*2.1.8.1 interne selecte communicatie*

*2.1.8.2 externe selecte communicatie*

*2.1.8.3 interne algemene communicatie*

*2.1.8.4 externe algemene communicatie*

2.1.9 ethiek

2.1.10 Overig/algemeen

*2.1.10.1 niets*

*2.1.10.2 landelijke afspraken*

*2.1.10.3 protocollen*

*2.1.10.4 overwegingen*

*2.1.10.5 ter kennisgeving aannemen*

*2.1.10.6 versnellen processen*

*2.1.10.7 bijlezen*

**2.2 binnen fase 2**

2.2.1 Infection control

*2.2.1.1 PBM*

2.2.1.1.1 voorraad

2.2.1.1.2 landelijke uniformiteit

*2.2.1.2 triage*

*2.2.1.3 casus definitie*

*2.2.1.4 ziektespecifiek*

2.2.2 Diagnosis

2.2.3 Patientenzorg

2.2.4 Staf/mankracht

*2.2.4.1 vragen/onrust*

2.2.4.2 inzet personeel

2.2.5 Institutionele capaciteit

2.2.6 derden

2.2.7 trainen

*2.2.7.1 opvang*

*2.2.7.2 overdracht*

*2.2.7.3 behandeling*

*2.2.7.4 monstername*

*2.2.7.5 overig/algemeen*

2.2.8 communicatie/informatie

*2.2.8.1 interne selecte*

2.2.8.1.1 beleid

2.2.8.1.2 poortspecialismen

*2.2.8.2 externe selecte*

2.2.8.2.1 met Huisartsen

2.2.8.2.1 met perifere ziekenhuizen

2.2.8.2.3 met academische ziekenhuizen

2.2.8.2.2 met Ambulancedienst

2.2.8.2.3 met regionaal publieke gezondheidszorg

2.2.8.2.4 met landelijke publieke gezondheidszorg

2.2.8.2.5. overig/algemeen

*2.2.8.3 interne algemene*

*2.2.8.4 externe algemene*

*2.2.8.5 overig*

2.2.9 ethiek

2.2.10 Overig/algemeen

*2.2.10.1 niets*

*2.2.10.2 landelijke afspraken*

*2.2.10.3 protocollen*

*2.2.10.4 overwegingen*

*2.2.10.5 informatie zoeken/opvragen*

2.2.11 Coördinatie

2.2.11.1 intern groep aanstellen die voorbereiding coordineert

**2.3 binnen fase 3**

2.3.1 Infection control

2.3.1.1 Triage

2.3.1.2. PBM

2.3.1.3 surveillance

2.3.1.4 Snelle isolatie

2.3.2 Diagnosis

2.3.3 Patienten zorg

2.3.4 Staf/mankracht

2.3.5 Institutionele capaciteit

2.3.6 derden

2.3.7 trainen/oefenen

2.3.7.1 opvang

2.3.7.2 overdracht

2.3.7.3 behandeling

2.3.7.4 PBM

2.3.7.5 omgaan met onrust

2.3.7.6 communicatie

2.3.8 communicatie/informatie

2.3.8.1 Specifiek intern

2.3.8.2 Specifiek extern

2.3.8.2.1 met Huisartsen

2.3.8.2.2 met perifere ziekenhuizen

2.3.8.2.3 met academische ziekenhuizen

2.3.8.2.4 met Ambulancedienst

2.3.8.2.5 met regionaal publieke gezondheidszorg

2.3.8.2.6 met landelijke publieke gezondheidszorg

2.3.8.2.7 overig/algemeen

2.3.8.4 Algemeen intern

2.3.8.5 Algemeen extern

Overig

2.3.9 ethiek

2.3.10 Overig/algemeen

2.3.10.1 niets

*2.3.10.2 landelijke afspraken*

*2.3.10.3 protocollen*

*2.3.10.4 overwegingen*

2.3.10.5 overig

**2.4 binnen fase 4**

2.4.1 Infection control

2.4.2 Diagnosis

2.4.3 Patiënten zorg

2.4.4 Staf/mankracht

2.4.5 Institutionele capaciteit

2.4.6 derden

2.4.7 trainen

2.4.8 communicatie/informatie

2.4.9 ethiek

2.4.10 Overig/algemeen

**2.5 afschaling**

**2.6 binnen onbekend fase**

Voorwaarden tussen fasen

**3.0 onbekende fase**

**3.1 voorwaarde fase 1**

**3.2 voorwaarde fase 2**

**3.3 voorwaarde fase 3**

**3.4 voorwaarde fase 4**

**3.5 voorwaarde afschaling**

**4. verschil opschaling grootschalige uitbraak**

**5. verantwoordelijkheid**

5.1 infectiepreventie eenheden binnen instelling

5.2 individuele experts binnen instelling

5.3 algemeen binnen instelling

**6. vraagt van…**

6.1 huisartsen

6.2 ambulancedienst

6.3 Perifere ziekenhuizen

6.4 academische ziekenhuizen

6.5 regionale publieke gezonheidszorg

6.6 landelijke publieke gezondheidszorg

6.7 algemeen

**Additional File 3A. Institutional preparedness activities per type of healthcare institution and per preparedness phase. (Dutch)**

|  | **Institutionele preparedness** | | | | |
| --- | --- | --- | --- | --- | --- |
|  | **Infectiepreventie** | **Diagnostiek** | **Patient’s care/cure** | **Personeel** | **Interne communicatie** |
| **Fase 3**  Uitvoerend | **GGD:**  Surveillance  Quarantaine  Vaccinatie | Diagnostiek (afh van type A-ziekte en beloop uitbraak) |  | Extra personeel / overuren door veelheid aan communcatie/vragen | interne communicatie Onrust bestrijden: vragenlijn bemand + Q&A op orde |
|  | **UMC:**  Triage | afhankelijk van pathogeen | Ethische afwegingen | Inzet extra personeel  Werktijden/ druk / stress  Morele ondersteuning |  |
|  | **Alg zkh:** |  |  |  | Intenser |
|  | **Amb:** |  |  |  |  |
|  | **Huisartsen:**  PBM  Afvalverwerking |  | Ethische afwegingen  Levensreddende handelingen |  |  |
| **Fase 2**  Explorerend | **GGD:**  Surveillance  Screening  Quarantaine  Protocollen controleren  Risicogroepen in de regio identificeren | Diagnostiek (afh van type A-ziekte en beloop uitbraak) |  | Extra personeel / overuren door veelheid aan communcatie/vragen | interne communicatie, vragenlijn bemand |
|  | **UMC:**  quarantaine | afhankelijk van pathogeen |  | onrust / angst | Subcommissie infectiecommissie  Personeel inlichten |
|  | **Alg ziekenhuizen:**  Triage z.n. aanscherpen  PBM juiste type op voorraad  Casusdefinitie |  |  | Extra monodisciplinaire training / oefening in het omkleden  Protocollen doorlopen voor opvang patiënt waaronder routes voor opvang en overdracht | VHK overleg  Evt. veranderende triage doorvoeren  Evt. geplande oefening communiceren |
|  | **Ambulancezorg:**  Screening  Casusdefinitie  Triage  PBM  Afvalverwerking |  | Ethische afwegingen,  Levensreddende handelingen | Morele ondersteuning  Training / Oefening / Simulatie  Onrust bestrijden |  |
|  | **Huisartsen:**  Screening/triage,  Casusdefinitie,  Type PBM |  | Ethische afwegingen,  Levensreddende handelingen: hangt moreel handelen af van het dreigingsniveau? | Huisartsen/triage voorlichten |  |
| **Fase 1** | **GGD:** Uitdiepen relatie uitbraak met de eigen regio | Diagnostiek (afh van type A-ziekte) |  | Monodisciplinair Trainen/oefenen/simulatie (afh van type A-ziekte)   - Aan- en uitkleden - Desinfectie | Interne communicatie |
|  | **UMC:**  Surveillance afh van pathogeen  Screening  Casusdefinitie  Omkleedprocedures  Afvalverwerking  Antivirale medicatie  PBM | Afh. van pathogeen | Prioritering / continuiteit van zorg  Levensreddende handelingen | training / oefening / simulatie  interne berichtgeving | poortspecialismen |
|  | **Alg zkh:** |  |  |  | Poortspecialismen informeren |
|  | **Amb:** |  |  |  |  |
|  | **HA:** |  |  |  |  |
| **Fase 0** |  |  |  |  |  |

**Additional file 3b. Collaborative Preparedness activities per preparedness phase.**

In bold the preparedness activities where the RIVM has been suggested as actor. Note: read from Phase yellow upwards, only new activities in the next phase were added. GP = general practitioner; PPE = personal protective equipment

|  | **Collaborative Preparedness** | | |
| --- | --- | --- | --- |
|  | **Information and/or Communication** | **Training and/or simulation** | **Coordination** |
| Phase red | Available treatment centers;  **The outbreak course;**  Transportation of patients over regional borders;  Contact tracing. | Training and/or simulation between academic hospitals and ambulance services. | **National Outbreak management team;**  **Referral towards available treatment centers;** |
| Phase orange | **Specific information materials;**  **Triage protocols;**  **Minimum type and number of personal protective equipment;**  Ethical considerations;  Regional synchronisation  Specific:   - Inform GP posts - Show involvement - Diminish unrest | Training and/or simulation between academic hospitals and ambulance services. | Available isolation rooms;  **Division of PPE / depot**  **National uniformity in minimum type of personal protective equipment;**  Informing GPs |
| Phase yellow | **Disease specific information;**  **Guidelines;**  **Outbreak**  **Case definition**  Degree of preparation;  **National capacity;**  National conventions in case of repatriation;  **Degree of menace;**  Regional synchronisation  Specific:   - Knowledge exchange |  | **Selection of treatment centers;**  **Task per chain partner;**  **National communication service;** |
|  |  |  |  |
